# Stability and repeatability of diffusion-weighted imaging (DWI) of normal pancreas on 5.0 Tesla magnetic resonance imaging (MRI)

**DOI:** 10.1038/s41598-023-38360-x

**Published:** 2023-07-24

**Authors:** Zhiyong Jiang, Wenbo Sun, Dan Xu, Hao Yu, Hao Mei, Xiaopeng Song, Haibo Xu

**Affiliations:** 1 Department of Radiology, Zhongnan Hospital of Wuhan University, 169 Donghu Rd, Wuchang District, Wuhan, Hubei China; 2 United Imaging Healthcare, Shanghai, China; 3 Wuhan Zhongke Industrial Research Institute of Medical Science, Wuhan, Hubei China

**Keywords:** Imaging, Preclinical research

## Abstract

To explore the stability and repeatability of diffusion-weighted imaging (DWI) of normal pancreas with different field of views (FOV) on 5.0 T magnetic resonance imaging (MRI) system. Twenty healthy subjects underwent two sessions of large FOV (lFOV) and reduced FOV (rFOV) DWI sequence scanning. Two radiologists measured the apparent diffusion coefficient (ADC) values and the signal-to-noise ratio (SNR) of the pancreatic head, body, and tail on DWI images, simultaneously, using a 5-point scale, evaluate the artifacts and image quality. One radiologist re-measured the ADC on DWI images again after a 4-week interval. The test-retest repeatability of two scan sessions were also evaluated. Intra-observer and inter-observer at lFOV and rFOV, the ADC values were not significantly different (*P* > 0.05), intraclass correlation coefficients (ICCs) and coefficient of variations were excellence (ICCs 0.85–0.99, CVs < 8.0%). The ADC values were lower with rFOV than lFOV DWI for the head, body, tail, and overall pancreas. The consistency of the two scan sessions were high. The high stability and repeatability of pancreas DWI has been confirmed at 5.0 T. Scan durations are reduced while resolution and image quality are improved with rFOV DWI, which is more preferable than lFOV for routine pancreas imaging.

## Introduction

Magnetic resonance imaging (MRI) is an essential tool for diagnosing pancreatic pathologies, including pancreatitis and pancreatic cancer^1,2^ However, imaging the pancreas by MRI is challenging due to its small size, respiratory motion and gas in the surrounding stomach and bowel^3^. This location of pancreas creates an uneven distribution of the B0 and B1+ fields, resulting in significant signal loss that makes improving the resolution and robustness of quantitative imaging in the pancreas a difficult task^4^.

Diffusion-weighted imaging (DWI) is a functional MRI sequence that can provide information on the diffusion of water molecules within biological tissues^5,6^. Apparent diffusion coefficient (ADC) is a quantitative measure derived from DWI, and has been increasingly used to evaluate and diagnose pancreatic pathologies^7^. In pancreatic imaging, ADC values have been found to be useful in differentiating between benign and malignant pancreatic lesions, as well as monitoring treatment response in pancreatic cancer patients^8–10^. One of the advantages of ADC value is its stability, as it is generally not affected by equipments or sequence parameters^11^. This stability is due to the fact that ADC value is a basic property of the tissue being imaged. However, ADC values may also be subject to variations due to biological factors, technical factors and measurement errors. For instance, increasing spatial resolution can lead to a rapid decrease in the signal-to-noise ratio (SNR) of DWI, which may result in a noise floor bias in ADC measures. Therefore, the spatial resolution of DWI is poor, which limited its application in pancreatic imaging.

Magnetic resonance reduced field of view (rFOV) imaging is a technique that overcomes resolution challenges and improves visualization of the pancreas^12^. The rFOV technique reduces the field of view in MRI imaging, resulting in higher spatial resolution and better imaging quality^13–16^. By reducing the field of view, rFOV-DWI imaging improves visualization of small structures, and minimizes signal loss caused by the inhomogeneous B1 + field distribution in the pancreas region^17^. Moreover, rFOV-DWI imaging targets specific regions of interest in the pancreas, improving detection and characterization of pancreatic pathologies^18^. In addition, high-field MRI at 3.0 Tesla (3.0 T) has become increasingly popular in clinical practice to improve the resolution of pancreatic MRI. Compared to traditional 1.5 Tesla (1.5 T) MRI, 3.0 T MRI offers several advantages in pancreatic imaging. One of the main advantages of 3.0 T MRI is its ability to provide better tissue contrast, which is particularly important in pancreatic imaging where it can be challenging to differentiate between pancreatic lesions and surrounding normal tissues. Another advantage of 3.0 T MRI is its higher SNR, which allows for better spatial resolution, improving detection and characterization of small pancreatic lesions and diagnostic accuracy. Combining high-field MRI with rFOV-DWI is an important diagnostic direction for the pancreas. Studies by Ma et al. and Kim et al. have shown that 3.0 T rFOV-DWI improves resolution by a factor of 2 compared to large-FOV (lFOV) DWI^12,19^. Donati et al. also reported higher image quality, anatomic detail, and lesion detection using rFOV-DWI versus lFOV-DWI of the pancreas on 3.0 T MRI^20^.

The clinical application of a newly developed 5.0 T magnetic resonance imaging (MRI) system in pancreas diagnostics has yet to be fully evaluated. Specifically, there is a lack of research on the quality, stability, and repeatability of lFOV and rFOV DWI on a 5.0 T MRI. Given that higher magnetic field strengths can lead to increased artifacts, particularly in the abdomen where the radiofrequency (RF) wavelength is shorter, it is crucial to investigate the repeatability of lFOV and rFOV DWI of pancreas at 5.0 T. Additionally, a comparison of the quality and consistency between lFOV and rFOV ADC is warranted. Our study aimed to assess the clinical utility of 5.0 T MRI for pancreas imaging and to explore the feasibility of using rFOV to enhance ADC image quality.

## Materials and method

### Subjects

Twenty healthy subjects participated in the prospective investigation and underwent MRI scanning of the pancreas between February 2022 and May 2022. This study was approved by the local Ethics Committee. All volunteers signed the informed consent form. Included if: (1) individuals aged 18–65; (2) no history of drug abuse, drug use one week before the exam, pancreatitis, diabetes, alcohol abuse, chronic liver diseases, hepatic steatosis, or abdominal surgery; (3) no contraindications for MRI (such as implanted pacemaker, metallic implant, and claustrophobia). Exclusion criteria: (1) incomplete DWI procedure due to any reasons, (2) poor image quality (with high motion artifacts) unsuitable for further imaging analysis.

### MRI scanning

The 5.0 T whole-body magnetic resonance scanning system (uMR Jupiter, United-Imaging Healthcare, Shanghai, China) was used for all MRI examinations in this study, using a flexible 24-channel body coil and the built-in 24-channel spine coil. Table 1 showed the scanning parameters.

**Table 1:** MR imaging sequence parameters.

	lFOV_T2	lFOV_DWI	rFOV_DWI
TR (ms)	~ 6537	~ 3833	~ 3555
TE (ms)	74.88	49.7	49.7
Flip angle	90°	90°	90°
Field of view (FOV)	300 × 380	300 × 380	120 × 240
In-plane resolution (mm^2)^	1.47 × 1.25	2.38 × 2.38	1.5 × 1.5
Matrix	204 × 304	126 × 160	80 × 160
Receiver bandwidth (kHz)	260	2300	2300
Slice thickness (mm)	6	4	4
No. of slices	24	16	16
Acceleration factor	2	2	2
Echo train length	13	51	48
B-values		0, 800	0, 800
Average times	1	1, 5	1,5
Total acquisition duration (s)	~252	~200	~125

For regular lFOV DWI scanning, the parameters were field of view (FOV): 300 × 380 mm^2^; voxel: 2.38 × 2.38 × 4 mm^3^; repetition time (TR): ~3833 ms; echo time (TE): 49.7 ms; bandwidth (BW): 2300 kHz; slice thickness: 4 mm; number of slices: 16; b values: 0 s/mm^2^ (average time = 1), and 800 s/mm^2^ (average time = 5); parallel imaging acceleration factor: 3; and scanning time: ~200 s.

For rFOV DWI scanning, the parameters were FOV: 120 × 240 mm^2^; voxel: 1.50 × 1.50 × 4 mm^3^; TR: 3555 ms; TE: 49.7 ms; BW: 2300 kHz; slice thickness: 4 mm; number of slices: 16; b values: 0 s/mm^2^ (average time = 1), and 800 s/mm^2^ (average time = 5); parallel imaging acceleration factor: 3; and scanning time: 125 s.

**Figure 1:**
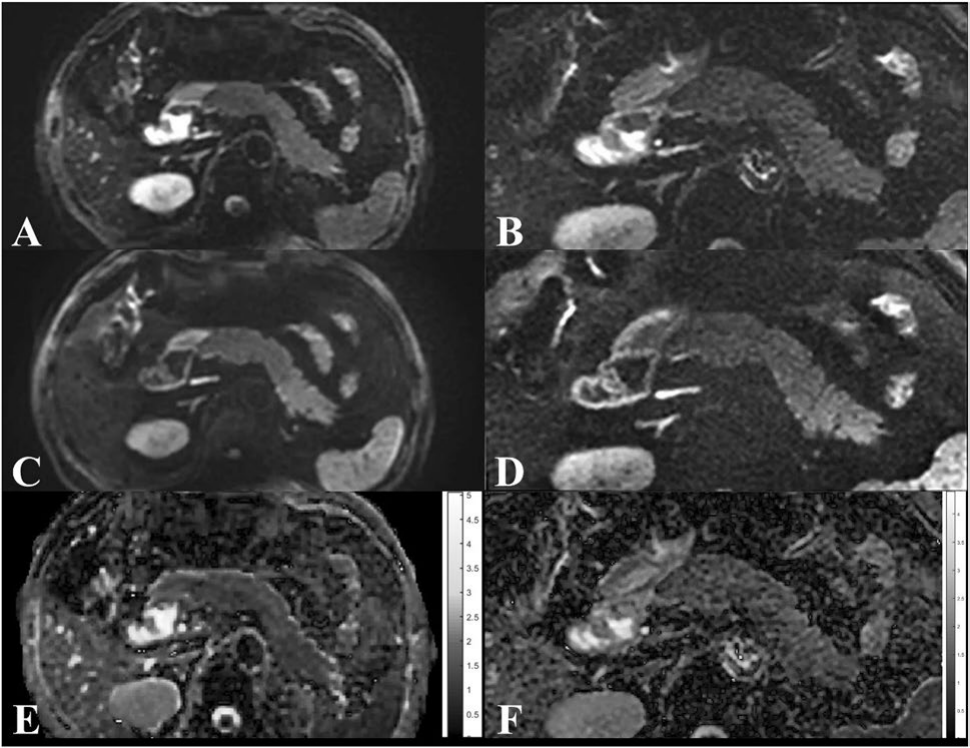
Male, 57 years old. lFOV: b0 (**A**), b800 (**C**), ADC map (**E**). rFOV: b0 (**B**), b800 (**D**), ADC map (**F**).

All subjects were fasted for at least 6 hours before the MRI examination. Each subject underwent two sessions of MRI scanning, after the first scanning session was completed, the second scanning session was performed after an interval of about 48 hours, and the sequence parameters and positioning for scanning were the same as the first session. For each scanning session, after shimming, each subject first underwent lFOV T2 scanning, and then lFOV DWI scanning, afterward, rFOV DWI scanning was performed and got the images (Fig. 1). The respiration-triggered scanning method (end-expiration triggering) was used.

### Data analysis

The MRI images of all participants were analyzed by the same two radiologists with 12 and 15 years of experience, respectively. The distribution of SNR and ADC values of manually selected regions of interest (ROIs) were examined. ROIs were selected from pancreatic head (from the margin of the right to the left superior mesenteric vein), pancreatic body (pancreas region from the left margin of the superior mesenteric vein to the left aorta), and tail (pancreas region from the left margin of the aorta to the hilus lienis). The pancreatic ducts, cystic lesions, or artifacts were avoided when choosing the ROIs. ROI was placed at least 6 mm from the pancreas margin to avoid the average volume effect.

For objectively assessing the signal-to-noise ratio (SNR) and ADC variability, the two radiologists delineated the ROIs of the pancreatic head, body, and tail on DWI images independently. One radiologist (observer 1) delineated the ROIs and re-measured the SNR and ADC on DWI images again after a 4-week interval. rFOV images do not include any areas outside of the body; therefore, air cannot be utilized in the calculation of SNR. SNR was calculated as the ratio between the average signal intensity and the standard deviation of the signal intensity manually placed circular ROIs. The average ADC value was acquired from the oval/round ROI on ADC images. The areas of ROI ranged from 50 to 100 mm^2^. The average ADC values of the pancreatic head, body, and tail were calculated as the overall ADC value of the pancreas.

The semi-quantitative grading system was used to evaluate image quality and artifact grade. The presence of artifacts was first assessed, and then two radiologists independently assessed the lFOV and rFOV DWI images using the 5-level criteria (5, no artifacts; 4, low amounts of artifacts not influencing the quality of diagnosis; 3, with artifacts that could reduce the quality of diagnosis; 2, with artifacts leading to the minimal information of diagnosis; and 1, could not be used for diagnosis). A subjective assessment of overall image quality was also performed (5, the best image quality; 4, high image quality for diagnosis; 3, images with limited diagnostic value/quality; 2, images provide only very limited information for diagnosis; and 1, could not be used for diagnosis).

### Statistical analysis

SPSS 26.0 was used for statistical analysis. The Shapiro–Wilk test was performed for the normality test. Continuous data with a normal distribution were described as means ± standard deviations and analyzed using the independent sample t-test. The paired t-test was used for the comparisons between the two measurements of ADC and SNR from the same investigator. Normality and Lognormality Tests are used to analyze the ADC value between rFOV and lFOV. Intra- and inter-observer variability and test–retest repeatability (the reprocucibility of two scanning sessions) of ADC measurements for each anatomical region of the pancreas and whole pancreas were analyzed by the Bland–Altman analysis, coefficient of variation (CV), and intra-class correlation coefficient (ICC). The Bland–Altman test and weighted κ coefficient was used to analyze the consistency of ADC between lFOV and rFOV. *P* < 0.05 was considered statistically significant.

### Ethics approval

The study was conducted in accordance with the Declaration of Helsinki, and approved by the institutional review board of Zhongnan Hospital, Wuhan University (No.2021095, December 2021). The subjects in the study provided written informed consent.

## Results

### Subject information

Twenty-two individuals underwent MRI scanning in this study. One was excluded due to severe artifacts, and another was excluded for not acquiring data in the scanning process. Finally, 20 volunteers were included in the analysis, of which 13 were males and 7 were females. The ages of the males and females were 48.9 ± 14.0 and 45.9 ± 15.5 years old, respectively.

### ADC pancreatic evaluation (head, body, tail)

**Figure 2:**
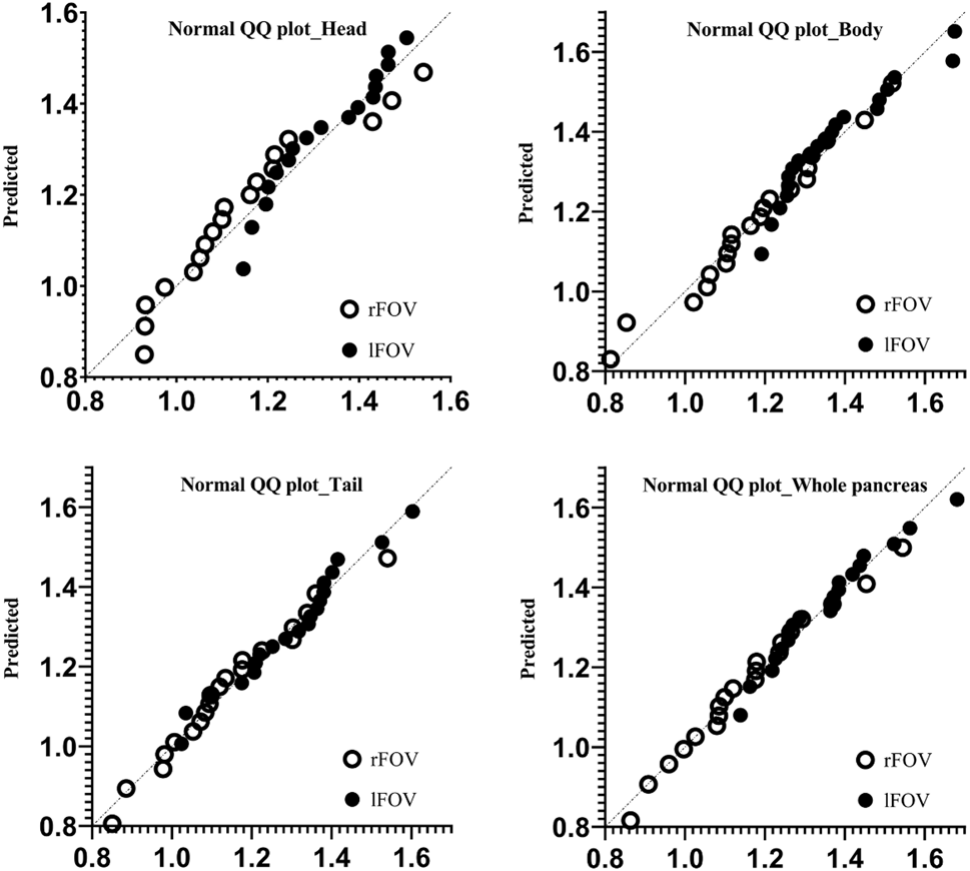
Normality and Lognormality Tests of QQ graph between lFOV and rFOV. In comparison to the body, the tail, and the whole pancreas, the normal distribution of ADC values in the head's rFOV and lFOV is somewhat inferior.

The ADC values of the pancreas, measured by two observers on a total of twenty subjects. Table 2 reported the mean and standard deviation for the head, body, and tail segments of the test and retest data. Tables 3 and 4 reported that of the intra-observer and inter-observer data There was no significant difference between the two ADC measurements made by the same observer for the whole or parts of the pancreas (*P* > 0.05), when comparing two different observers, significant differences in ADCs were observed for the tail in lFOV (*P* = 0.014), the body in rFOV (*P* = 0.042). The normal distribution of ADCs for fFOV and rFOV is demonstrated in Fig. 2.

**Table 2:** Comparing the pancreatic ADC values (× 10^–3^ mm^2^/s) of lFOV and rFOV various anatomic locations for Test-Retest of two scan swssions.

	Test	Retest	P	ICC	CV
lFOV					
Head	1.374 ± 0.195	1.381 ± 0.199	0.553	0.959	4.0%
Body	1.352 ± 0.151	1.372 ± 0.157	0.268	0.879	5.1%
Tail	1.289 ± 0.149	1.282 ± 0.162	0.337	0.884	2.9%
Whole pancreas	1.338 ± 0.145	1.345 ± 0.151	0.497	0.898	3.2%
rFOV					
Head	1.132 ± 0.222	1.186 ± 0.231	0.109	0.799	12.4%
Body	1.173 ± 0.222	1.179 ± 0.194	0.851	0.839	11.8%
Tail	1.124 ± 0.175	1.154 ± 0.188	0.314	0.789	11.2%
Whole pancreas	1.143 ± 0.179	1.173 ± 0.189	0.267	0.817	9.5%

**Table 3:** Comparing the pancreatic ADC values (× 10^–3^ mm^2^/s) of lFOV and rFOV at various anatomic locations for intra-observer concordance in Observer 1.

	1st	2nd	*P* value	ICC	CV
lFOV					
Head	1.371 ± 0.191	1.381 ± 0.175	0.302	0.971	3.0%
Body	1.354 ± 0.184	1.345 ± 0.163	0.299	0.954	2.8%
Tail	1.262 ± 0.154	1.298 ± 0.149	0.662	0.981	2.3%
Whole pancreas	1.329 ± 0.147	1.350 ± 0.138	0.207	0.976	2.1%
rFOV					
Head	1.164 ± 0.186	1.176 ± 0.146	0.883	0.987	1.9%
Body	1.142 ± 0.222	1.154 ± 0.212	0.179	0.992	2.4%
Tail	1.116 ± 0.176	1.138 ± 0.169	0.867	0.908	6.5%
Whole pancreas	1.141 ± 0.180	1.136 ± 0.169	0.604	0.902	6.4%

**Table 4:** Comparing the pancreatic ADC values (× 10^–3^ mm^2^/s) of lFOV and rFOV at various anatomic locations for inter-observer concordance.

	Observer1	Observer2	*P* value	ICC	CV
lFOV					
Head	1.371 ± 0.167	1.381 ± 0.174	0.284	0.968	3.2%
Body	1.359 ± 0.135	1.373 ± 0.142	0.172	0.946	4.2%
Tail	1.295 ± 0.459	1.278 ± 0.148	0.014	0.981	2.5%
Whole pancreas	1.342 ± 0.132	1.335 ± 0.137	0.351	0.974	2.3%
rFOV					
Head	1.175 ± 0.172	1.166 ± 0.167	0.099	0.991	2.5%
Body	1.146 ± 0.208	1.159 ± 0.215	0.042	0.992	2.5%
Tail	1.142 ± 0.159	1.120 ± 0.171	0.216	0.899	6.8%
Whole pancreas	1.145 ± 0.160	1.158 ± 0.174	0.538	0.846	8.0%

### Test–retest repeatability

**Figure 3:**
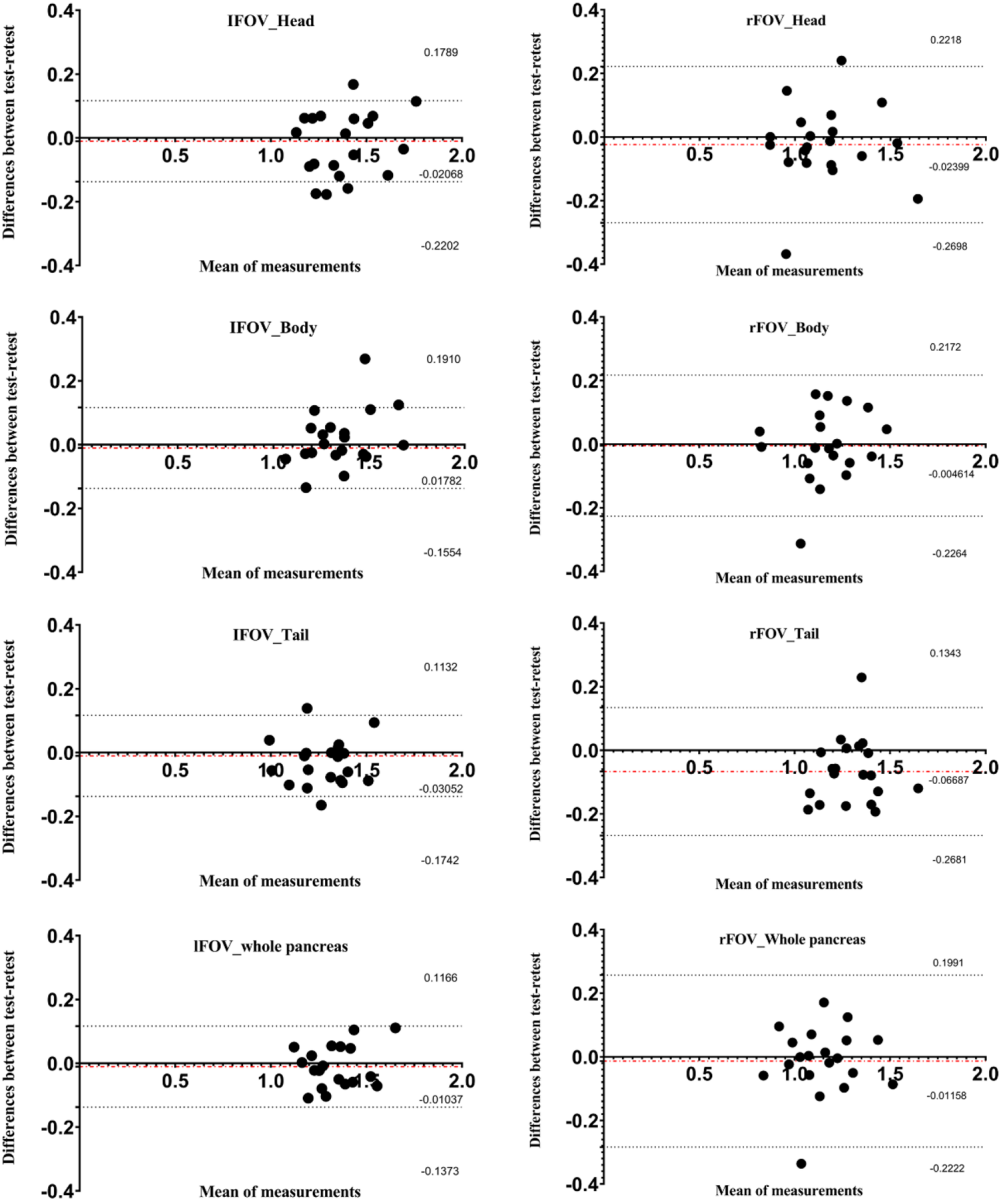
Test–retest reproducibility of ADC values for the 3 pancreatic segments and the whole pancreas. Bland–Altman plots of differences in test-ADC measurements (y-axis) against the retest-ADC measurement (x-axis), with a mean absolute difference (bias) (red dashed lines) and 95% confidence intervals of the mean difference (limits of agreement, LOA) (black dashed lines).

Test–retest/Scan-rescan repeatability ADC in the head, body, and tail segments were been given by Table 2. ADC repeatability showed excellent in lFOV (ICC 0.884–0.995, CV 2.9–5.1%), ADC repeatability showed good in rFOV (ICC 0.789–0.839, CV 9.5–12.4%). Corresponding Bland–Altman plots are shown in Fig. 3.

### Intraobserver and interobserver repeatability

**Figure 4:**
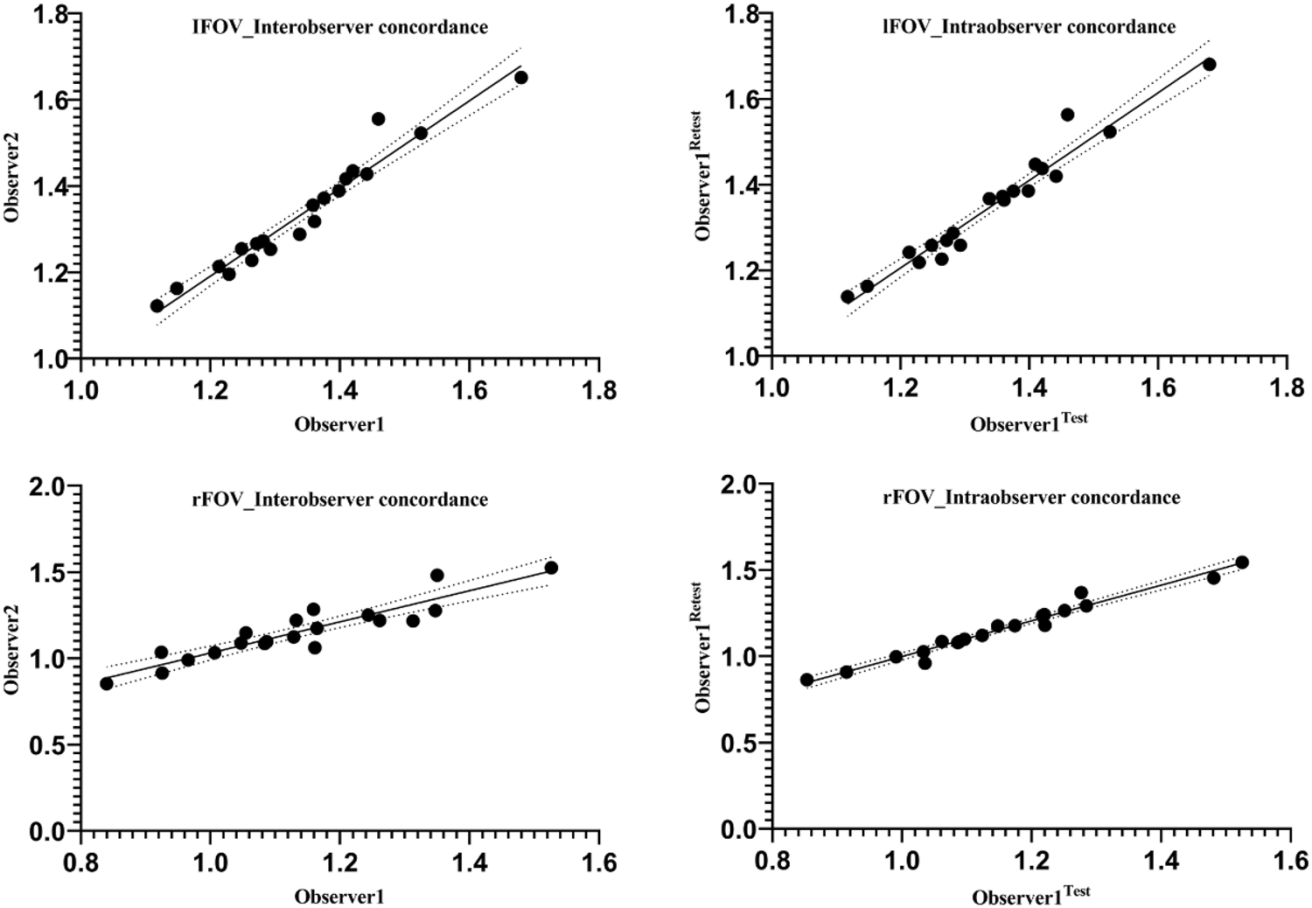
Scatter plot of intra-observer and inter-observer correlations. Results show excellent inter-observer and intra-observer agreement both in lFOV and rFOV.

The ADCs in lFOV and rFOV showed excellent intra-observer reproducibility and inter-observer agreement (with ICCs 0.846–0.992 and CVs 2.3–8.0% inter-observer, with ICCs 0.902–0.992 and CVs 1.9–6.5% intra-observer) for the head, body, tail, and whole pancreas in lFOV and rFOV, respectively (Tables 3, 4 and Fig. 4).

### SNR and subjective evaluation

**Figure 5:**
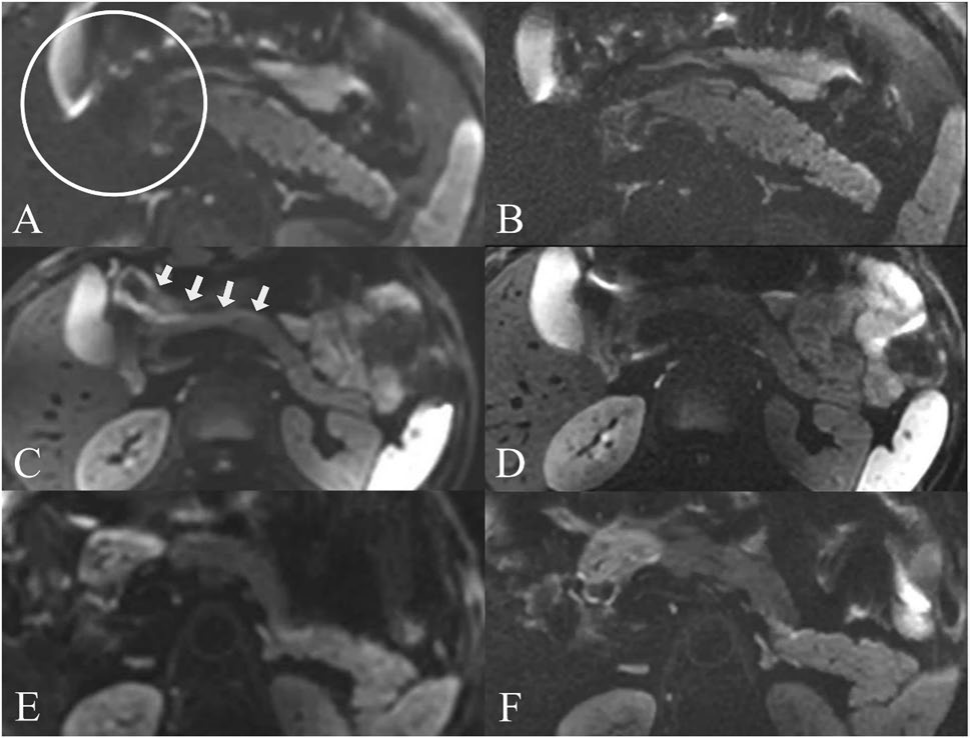
b800 of lFOV (**A**, **C**, **E**), b800 of rFOV (**B**, **D**, **F**). According to the image, rFOV images have a greater resolution and more clear contours than lFOV images. In **A**, **B**, on the b800 DWI image of lFOV, as depicted by the white solid circle, the head of the pancreas is obscured by the adjacent gallbladder and surrounding intestines, and the partial display is not clear. In **C**, **D**, the position shown by the white pointed head, the pancreas appears pseudo-diffusion-restricted in lFOV due to the convolution effect of the surrounding intestine, this effect disappears in rFOV DWI. In **E**, **F**, demonstrate that the diffuse look of the body and tail of the pancreas on DWI images is distinct, because of that the head, body, and tail of the pancreas have distinct cellular compositions.

SNR values were around 1.13–1.17 times higher with lFOV than rFOV for the head (20.286 ± 3.907 vs. 17.924 ± 4.485 dB), the body (20.157 ± 5.710 vs. 17.842 ± 4.908 dB), and the tail (19.418 ± 5.057 vs. 16.626 ± 3.903 dB) in Table 5. Two observers' subjective evaluations indicate that rFOV is considerably superior to lFOV in terms of artifact evaluation and image quality evaluation (Table 6 and Fig. 5).

**Table 5:** SNR comparisons of lFOV and rFOV.

	lFOV	rFOV	*P* value
SNR head	20.286 ± 3.907	17.924 ± 4.485	0.005
SNR body	20.157 ± 5.710	17.842 ± 4.908	0.057
SNR tail	19.418 ± 5.057	16.626 ± 3.903	0.019

**Table 6:** Image artifacts and overall image quality scores.

Parameter	lFOV	rFOV	*P* value
Image artifacts			
Observer 1	3.450 ± 0.510	4.100 ± 0.447	0.031
Observer 2	3.850 ± 0.587	4.600 ± 0.503	0.001
ICC	0.588	0.421	N/A
Overall image quality			
Observer 1	3.500 ± 0.607	4.250 ± 0.550	0.000
Observer 2	3.750 ± 0.444	4.550 ± 0.510	0.002
ICC	0.488	0.422	N/A

## Discussion

The aim of this study was to assess the stability and repeatability of lFOV and rFOV DWI of pancreas at 5.0 T by examining and comparing ADC values. In test-retest experiments, ADC repeatability showed good to excellent (ICC 0.789–0.995, CV 2.9%–12.4%). Intra-observer ADC consistency was excellent (ICC 0.902–0.992, CV 1.9–6.5%), while inter-observer ADC consistency was also excellent (ICC 0.846–0.992, CV 2.3–8.0%). Additionally, we found that the SNR of the rFOV was comparable to that of the lFOV, but the rFOV was superior to the lFOV in terms of subjective evaluation and had a shorter scanning time. These findings suggest that rFOV is more favorable for routine pancreas diagnostic imaging on a 5.0 T MRI.

The apparent diffusion coefficient (ADC) is a crucial parameter for evaluating microstructural changes in tissues, organs, and lesions^21,22^. Evaluating the repeatability of ADC values in diffusion-weighted imaging (DWI) is clinically important, as patients may undergo multiple DWI examinations to monitor a treatment effect or follow up on a lesion^23^. Ensuring low intra- and inter-observer ADC CV is a desirable goal. Although ADC repeatability has been examined for various organs, including the breast, lung, and liver, data are limited for the pancreas^24–27^. Our study found that the intra- and inter-observer ADC CVs were comparable to a previous study that reported mean ADC CVs of 10.6% for the whole pancreas in 3.0 T DWI examinations^28^. Another study reported CVs of 9%, 8%, and 8% for the head, body, and tail of the pancreas^29^. Additionally, two studies reported good ADC reproducibility of the pancreas at 1.5 and 3.0 T^30,31^. However, discrepancies between studies may be due to differences in DWI sequence parameters, including the selection of b-values^32^, as well as respiration compensation acquisition and post-processing methods, which can affect ADC values^33^.

The 5.0 T MRI is a novel system that has recently become available for clinical diagnosis. However, there are few studies that have investigated 5.0 T rFOV-DWI. Zhang et al. has shown no significant differences in lFOV-DWI ADC values between 3.0 T and 5.0 T magnets in nine volunteers^34^. The MRI protocol in Zhang et al. was similar to that used in our study, and their whole-pancreas ADC values (1.394 ± 0.130 × 10^-3^ s/mm^2^) were comparable to those in our study (1.342 ± 0.132 × 10^–3^ mm^2^/s), and in previous 3.0 T studies (1.416 ± 0.175 × 10^–3^ mm^2^/s), while another study by Zheng et al. showed significant higher ADC values (1.671 ± 0.226 × 10^-3^ s/mm^2^) in pancreas than our findings^35^. This could be due to a different choice of spatial resolution in sequence parameters and a partial volume effect.

In our study, the ADC values of the pancreatic tail were found to be the lowest, which is consistent with previous reports^29,36^. Due to the lower ADC values, even small variations can lead to higher CV compared to higher ADC values. The pancreas is approximately 15 cm long and located behind the stomach, which may lead to gradient nonlinearity and affect ADC values^37,38^. Additionally, previous quality control studies have reported significant ADC errors for scanning areas away from the magnet isocenter^39^. Anatomical investigations have revealed that the head, body, and tail of the pancreas have distinct cellular compositions^40^. These factors may all contribute to higher CVs of the pancreatic tail.

In addition, our study found that the ADC values measured with rFOV were lower than those with lFOV, which is in line with several earlier studies^15,17^. This phenomenon may be attributable to various factors. Firstly, the resolution of the rFOV DWI series is substantially higher than that of the lFOV sequence, which reduces the number of hydrogen spins contained in a single voxel, resulting in lower transverse magnetization and less information being gathered^41^. Secondly, due to the smaller FOV, certain external and adjacent tissue and organ signals outside of the FOV are filtered out, whereas these signals may contribute to higher ADC values in a large FOV DWI. Lastly, the modalities of RF excitation for rFOV and lFOV DWI sequences are different, and rFOV DWI may be more sensitive to abdominal movement and other physiological noise.

### Limitations

There are several limitations to the current study. Firstly, the sample size was small, and all participants were healthy. Thus, it is unclear whether the results can be generalized to pancreas lesions. Secondly, it would have been preferable to include several repeated assessments instead of just two repeated examinations to accurately assess the repeatability of the measurements. Thirdly, the study only demonstrated short-term reproducibility of DWI on ultra-high field MRI, which limits the ability to evaluate all potential factors of long-term variability.

## Conclusions

Our study demonstrated high stability, repeatability, and consistency of ADC values for the pancreas in both lFOV and rFOV at 5.0 T DWI sequences. Moreover, rFOV DWI showed improved resolution and image quality while reducing scan duration. Therefore, 5.0 T DWI may serve as a reliable tool for clinical diagnosis of pancreas diseases, and rFOV DWI is a feasible quantitative imaging tool for investigating lesions and changes of the pancreas in clinical settings.

## Data Availability

The datasets used and/or analysed during the current study available from the corresponding author on reasonable request.

## Abbreviations

DWI: Diffusion-weighted image
ADC: Apparent diffusion coefficient
lFOV: Large field of view
rFOV: Reduced field of view
MRI: Magnetic resonance imaging
SNR: Signal-to-noise ratio
